# African-American mitochondrial DNAs often match mtDNAs found in multiple African ethnic groups

**DOI:** 10.1186/1741-7007-4-34

**Published:** 2006-10-12

**Authors:** Bert Ely, Jamie Lee Wilson, Fatimah Jackson, Bruce A Jackson

**Affiliations:** 1 Department of Biological Sciences, University of South Carolina, Columbia, South Carolina, 29208, USA; 2 Biomedical Engineering and Biotechnology Program, University of Massachusetts, Lowell, MA 01854, USA; 3 Department of Anthropology, University of Maryland, College Park, MD 20742, USA

## Abstract

**Background:**

Mitochondrial DNA (mtDNA) haplotypes have become popular tools for tracing maternal ancestry, and several companies offer this service to the general public. Numerous studies have demonstrated that human mtDNA haplotypes can be used with confidence to identify the continent where the haplotype originated. Ideally, mtDNA haplotypes could also be used to identify a particular country or ethnic group from which the maternal ancestor emanated. However, the geographic distribution of mtDNA haplotypes is greatly influenced by the movement of both individuals and population groups. Consequently, common mtDNA haplotypes are shared among multiple ethnic groups. We have studied the distribution of mtDNA haplotypes among West African ethnic groups to determine how often mtDNA haplotypes can be used to reconnect Americans of African descent to a country or ethnic group of a maternal African ancestor. The nucleotide sequence of the mtDNA hypervariable segment I (HVS-I) usually provides sufficient information to assign a particular mtDNA to the proper haplogroup, and it contains most of the variation that is available to distinguish a particular mtDNA haplotype from closely related haplotypes. In this study, samples of general African-American and specific Gullah/Geechee HVS-I haplotypes were compared with two databases of HVS-I haplotypes from sub-Saharan Africa, and the incidence of perfect matches recorded for each sample.

**Results:**

When two independent African-American samples were analyzed, more than half of the sampled HVS-I mtDNA haplotypes exactly matched common haplotypes that were shared among multiple African ethnic groups. Another 40% did not match any sequence in the database, and fewer than 10% were an exact match to a sequence from a single African ethnic group. Differences in the regional distribution of haplotypes were observed in the African database, and the African-American haplotypes were more likely to match haplotypes found in ethnic groups from West or West Central Africa than those found in eastern or southern Africa. Fewer than 14% of the African-American mtDNA sequences matched sequences from only West Africa or only West Central Africa.

**Conclusion:**

Our database of sub-Saharan mtDNA sequences includes the most common haplotypes that are shared among ethnic groups from multiple regions of Africa. These common haplotypes have been found in half of all sub-Saharan Africans. More than 60% of the remaining haplotypes differ from the common haplotypes at a single nucleotide position in the HVS-I region, and they are likely to occur at varying frequencies within sub-Saharan Africa. However, the finding that 40% of the African-American mtDNAs analyzed had no match in the database indicates that only a small fraction of the total number of African haplotypes has been identified. In addition, the finding that fewer than 10% of African-American mtDNAs matched mtDNA sequences from a single African region suggests that few African Americans might be able to trace their mtDNA lineages to a particular region of Africa, and even fewer will be able to trace their mtDNA to a single ethnic group. However, no firm conclusions should be made until a much larger database is available. It is clear, however, that when identical mtDNA haplotypes are shared among many ethnic groups from different parts of Africa, it is impossible to determine which single ethnic group was the source of a particular maternal ancestor based on the mtDNA sequence.

## Background

**Figure 1:**
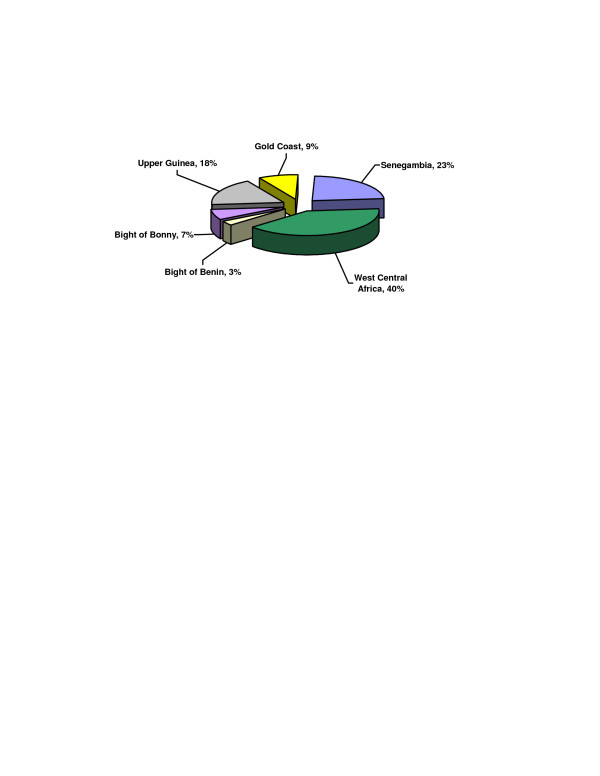
Proportions of enslaved Africans brought to historic Carolina coast ports from the 17th to 19th centuries CE (from Jackson, 2004 [2]).

The Atlantic slave trade resulted in the forced migration of an estimated 11 million Africans to the Americas. Only 9 million are thought to have survived the passage, and many more died in the early years of captivity. Historical accounts indicate that virtually all enslaved Africans brought to North America came from either West or West Central Africa. A recent comparison of mtDNA sequences from 1148 African Americans living in the US with a database of African mtDNA sequences showed that more than 55% of the US lineages have a West African ancestor, while fewer than 41% came from West Central or South West Africa [1]. In North America, different constellations of African groups were brought to various staging areas [2]. Among the important staging areas for the arrival and distribution of enslaved Africans were the ports of Savannah, GA and Charleston, SC. Estimates of the origin of enslaved Africans received at these sites are presented in Figure 1, with the largest African regional contributions coming from West Central Africa (40%; contemporary Angola, the Congos, Equatorial Guinea, and Gabon), and the West African regions of Senegambia (23%; contemporary Senegal, Gambia, and northern Guinea), and Upper Guinea (18%; contemporary Guinea and Sierra Leone and northwestern Liberia). Africans in the Carolina coast region were intentionally mixed to reduce the possibilities for successful revolts and to facilitate their assimilation into plantation-slave society. The contemporary Gullah/Geechee culture emerged from these Africans.

Because mitochondrial DNA (mtDNA) is passed from mother to daughter with few, if any, changes occurring over many generations, it is possible to compare contemporary African-American mtDNA haplotypes with contemporary mtDNA haplotypes in a worldwide database to obtain information about the ancestral origins of these mtDNAs. In such a comparison, continent-specific haplotypes are readily observed, and the assignment of mtDNAs to continent of origin is relatively straightforward. The more difficult task is to tie particular mtDNA haplotypes to specific geographical regions and ethnic groups within a continent. This task is particularly difficult for Africa, as there is more genetic diversity among Africans than among people from any other continent and because humanity has resided in Africa longer than anywhere else.

Comparisons of individual mtDNA haplotypes could be used to identify a geographical region, particular country, or even an ethnic group from which a maternal ancestor emanated. However, the geographic distribution of mtDNA haplotypes is greatly influenced by the migration of individuals or population groups. These movements often result in the assimilation of people from other ethnic groups. Intermarriage also causes mtDNA haplotypes to move from one ethnic group to another. Over time, mtDNA haplotypes that originated in a single ethnic group are distributed among many ethnic groups. Despite these complications, mtDNA analyses for the purposes of ancestry reconstruction are increasing in popularity. Many people have had their mtDNA tested with the hope that the test will match their DNA to an mtDNA haplotype found in a particular ethnic group. For African Americans, who have been disenfranchised from their specific African roots, such a test might provide a clue about the ethnic group or country in Africa where one of their maternal ancestors originated. However, if identical mtDNA haplotypes are shared among many ethnic groups from different parts of Africa, it would be impossible to use DNA sequence information to determine which single ethnic group was the source of a particular maternal ancestor. To date, there are no published assessments that provide quantitative information about how often African-American mtDNAs are exact matches to multiple African ethnic groups. Therefore, we decided to compare samples of Carolina coast and other African-American mtDNAs to a database of sub-Saharan African mtDNAs to generate such an assessment.

## Results

### Database characterization

We assembled a database of 3645 mtDNA HVS-I sequences from the published literature and 80 additional sequences from our own (unpublished) studies of ethnic groups in Mali to generate a database of 3725 sequences. Only sequences from sub-Saharan Africa were included in the database, because North African mtDNAs are quite different from sub-Saharan mtDNAs [1] and few North American slaves are thought to have come from North African countries. Within the sub-Saharan database, more than 50% of the sequences were identical to a sequence from at least one other ethnic group. The remaining sequences either occurred multiple times within a single ethnic group or occurred only once in the database.

**Figure 2:**
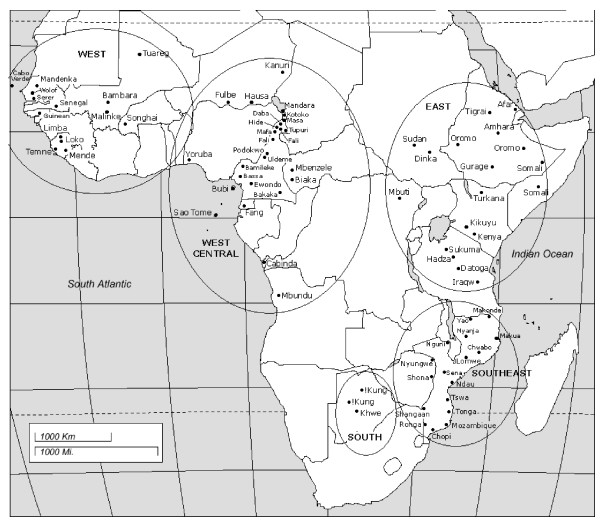
Map depicting the geographic locations and the regional groupings of the population samples used in this study.

To provide a regional analysis of the database, samples were assigned to geographic regions as shown in Table 1 and Figure 2, and the percentages of within-region and among-region matches were determined. The West African region contributed 1528 (41%) of the sequences (Table 2). The sizes of the other regional groups ranged from 127 to 995. Overall, 40% of the sequences were present only once in the database or were found multiple times within a single ethnic group. In contrast, 24% of sequences were found in multiple ethnic groups from at least three geographical regions.

Two of the regional groupings, East and South, had an excess of sequences that were found in a single ethnic group, and a corresponding deficit of matches to sequences from multiple regions. This result is consistent with the idea that these two regions are dominated by samples that have low levels of the mtDNA haplotypes that are characteristic of the Bantu [4,5]. In contrast, the majority of mtDNA sequences from Mozambique in the Southeast region match sequences from multiple regions, and only a small percentage of these sequences are unique to ethnic groups from Mozambique, perhaps reflecting the fact that only Bantu speakers were sampled [5,6]. In support of this idea, most matches that include sequences from only two regions involve the West Central region that is believed to have been the original Bantu homeland [7].

### Comparison of African-American samples with the sub-Saharan databases

Two African-American samples, a sample of African Americans who self-identified as Gullah/Geechee and a sample of African-American DNAs obtained from the Armed Forces DNA Identification Laboratory (AFDIL), were compared with both the original and the expanded databases to provide a sense of how increasing the database size impacts the distribution of exact matches. The Gullah/Geechee people are an African-American microethnic group residing in the Georgia/South Carolina Lowcountry and coastal islands whose numbers are now estimated between 200,000 and 500,000 in the Sea Islands of South Carolina, Georgia, North Florida, and beyond [8]. Gullah/Geechee language and culture include unique practices and artefacts (e.g., coiled basketry, *Brer Rabbit *stories, praise houses) including a distinct linguistic style with roots among the Mende peoples of Sierra Leone, West Africa. When a sample of 74 Gullah/Geechee mtDNA sequences was compared with the sub-Saharan database, approximately half of the mtDNAs were identical to two or more mtDNAs in the database and only seven mtDNAs matched mtDNAs from a single ethnic group (Table 3). The remaining 28 mtDNAs were not identical to any sequence in the expanded database.

Similar results were obtained when the 97 African-American AFDIL mtDNAs were compared with the databases. Approximately half (49) of the mtDNAs were identical to multiple sequences in the original database (Table 3). As with the Gullah/Geechee sample, fewer than 10% of the sequences matched a sequence from a single ethnic group, and 40% of the sequences did not have any perfect match in the database.

When the unmatched AFDIL and Gullah/Geechee mtDNAs were combined and analyzed further, 63% differed from a database sequence at a single nucleotide position (Table 4). Nearly three-quarters of these imperfect matches were to sequences that were found in multiple ethnic groups. Thus, most of the imperfect matches appear to be derived from the common haplotypes by a single mutational event.

### Geographical distribution of database matches

The majority of African-American mtDNAs that were identical to database mtDNAs matched mtDNAs from ethnic groups that were scattered throughout sub-Saharan Africa. However, 41% of the Gullah/Geechee and 37% of the AFDIL mtDNAs that matched database sequences were identical to mtDNAs found only in western (West plus West Central) Africa (Table 5). Only one Gullah/Geechee mtDNA and one AFDIL mtDNA matched mtDNAs that are found exclusively in eastern Africa in the sub-Saharan database. This distribution of matches is consistent with the historical information that most North American slaves were originally from western Africa. Most of the single region matches to both the Gullah/Geechee and the AFDIL mtDNAs occurred with West African samples (Table 6). This result is consistent with the historical records indicating that West Africa was a major source of American slaves, but it also probably reflects the fact that the West African samples made up 41% of the expanded database. Surprisingly, five AFDIL mtDNAs matched only mtDNAs from the two Angolan samples that make up 4% of the database. This result is consistent with historical records indicating that a large proportion of the enslaved Africans brought to the Americas came from the West Central African region of Angola/Congo region, and suggests that ethnic groups in this region of Africa need to be sampled more extensively.

### Language group comparisons

Considering Africa's geographical size and population density, and the duration of human residence on this continent, linguistic diversity at the taxonomic level of family is amazing low. This low level of linguistic diversity is probably the consequence of protracted mobility and interaction among Africa's indigenous groups, facilitated by the longstanding presence of such organized political-social units as kingdoms and empires and such sociocultural practices as polygamy.

Among the AFDIL sequences with more than five matches to various African ethnic groups, most language diversity was within the various subfamilies of the Niger-Congo family. These subfamiliesinclude Atlantic Congo (e.g., the ethnic groups Fula, Yoruba, Wolof, Balanta) and Mande (e.g., the ethnic groups Mandingo, Mende, Bambara). However, in some of the sequence matches, different linguistic families were represented altogether, including the Afro-Asiatic (e.g., the Tuareg ethnic group) and Nilo-Saharan (e.g., the Dinka ethnic group) families, along with members of the Niger-Congo family.

The most extensive pan-African haplotype (16189 16192 16223 16278 16294 16309 16390) is in the L2a1 haplogroup. This sequence is observed in West Africa among the Niger-Congo family including the Malinke, Wolof, and others; in North Africa among the Afro-Asiatic family including the Hausa and others; in Central Africa among the Niger-Congo family including the Bamileke and others; in South Africa among the Khoisan family including the Khwe and the Niger-Congo family Bantu speakers; and in East Africa among the Niger-Congo family Kikuyu. Closely related variants are observed among the Afro-Asiatic family including the Tuareg in North and West Africa and among the East African Nilo-Saharan family Dinka. Thus, identical mitochondrial haplotypes are often shared among ethnic groups with considerable language diversity.

## Discussion

Because only a small fraction of the sub-Saharan African ethnic groups have been sampled, and there are parts of sub-Saharan Africa that are poorly represented in our database (Figure 2), our database cannot be considered a representative subset of the sub-Saharan mtDNA gene pool. Nevertheless, it is clear that a much larger database is needed since 40% of the African-American samples analyzed have no exact match in our database. The extensive sharing of mtDNA haplotypes among ethnic groups from different regions of Africa is consistent with the historical evidence of extensive migration and mixing of African ethnic groups. Indeed, the well-documented Bantu migrations appear to have had a major impact [4], as have the formation of the historic empires and kingdoms of the region (such as the historic empires of Ghana, Mali, and the Songhai, Bakongo, and Ashanti Kingdoms). Despite the limitations of our database of sub-Saharan mtDNA sequences, it is likely that we have identified the most common haplotypes found in this region. Some are found throughout the region that includes the Bantu migrations, and others are found primarily in either the western or the eastern parts of the continent. We intend to continue to increase the size of our database, because a significantly larger database would provide more information about haplotypes that are present at lower frequencies than the most common haplotypes. Some of these lower-frequency haplotypes are likely to be shared among widely distributed ethnic groups, while others may have a more localized distribution.

Another way to assess our sub-Saharan mtDNA database would be to see how well African-American mtDNAs match database sequences. Historical accounts of the trans-Atlantic slave trade indicate that most North American slaves came from the western coast of Africa, including the geographical regions from present-day Angola to Senegal. When African-American mitochondrial DNA HVS-I sequences were studied, nearly half were identical to those from two or more African ethnic groups in our expanded database. Furthermore, the average number of perfect matches per matching African-American mtDNA increased from 3.6 different ethnic groups to 6.1 different ethnic groups when the size of the database was increased by 53% to its present size of 3725 sequences. These results reflect the fact that approximately half the mtDNA sequences in our sub-Saharan database are shared by members of three or more ethnic groups.

In both of the African-American samples, approximately 40% of the mtDNA sequences did not match any sequence in any other ethnic group (Table 3). However, more than half of these sequences differed from multiple database sequences at a single position (Table 4). Because it is unlikely that more than a few of these differences result from new mutations that occurred in North America or that more than a few lineages went extinct in Africa after being introduced to the new world, this result suggests that only a small fraction of the mtDNA diversity present in sub-Saharan Africa has been sampled, and that much of the unsampled diversity is due to single mutations that have occurred in the common haplotypes.

Many African Americans are interested in learning more about their African roots and are willing to pay to have their mtDNA analyzed in the hope that it will match DNA from a particular African ethnic group. However, as more than half of the mtDNA sequences in the African database are identical to sequences from other ethnic groups, African-American mtDNAs will be much more likely to match sequences from multiple ethnic groups than sequences from a single ethnic group. When this result is coupled with the fact that 40% of African-American mtDNAs did not match any sequence in the database, it is clear that matches to a single African ethnic group will not be the outcome for most African Americans, and even when a match to a single ethnic group is obtained, multiple matches may occur in a larger database. Furthermore, for the typical African American, the maternal ancestor who was the source of the mtDNA was just one of hundreds of enslaved African ancestors. In fact, it likely that there has been more mixing of African ethnic groups in the Americas than has ever occurred elsewhere. Thus, the ancestors of virtually all contemporary African Americans came from a large number of ethnic groups located throughout the region from Senegal to Angola.

## Conclusion

Half of the sub-Saharan mtDNA sequences in our database are common haplotypes that are shared among ethnic groups from multiple regions of sub-Saharan Africa. The finding that fewer than 10% of African-American mtDNAs matched mtDNA sequences from a single African region suggests that as few as one in nine African Americans may be able to trace their mtDNA lineage to a particular region of Africa. However, no firm conclusions should be made until a much larger database is available. It is clear, however, that nearly half of contemporary African-American mtDNAs are identical to African haplotypes that are found in multiple ethnic groups throughout sub-Saharan Africa. For these mtDNAs, it is impossible to use only mtDNA sequence information to determine which single ethnic group was the source of the maternal ancestor.

## Methods

### African-American samples

A sample of 78 African Americans who self-identified as Gullah/Geechee was generated by our laboratories from unrelated people sampled in the coastal areas of South Carolina and Georgia using either cheek swabs or mouthwash to collect buccal cells. DNA was isolated using a BuccalAmp DNA Extraction Kit (Epicentre, Madison, WI) for the cheek swabs or a DNAzol procedure (Molecular Research Center, Cincinnati, OH) for the mouthwash samples. The HVS-I region was amplified and sequenced as described previously [3]. Those mtDNAs with non-African haplotypes, three with Native American haplotypes (two haplotype B and 1 haplotype A2) and one with European mtDNA (haplotype H) were excluded from further analysis (Table 9). A second sample of 104 African-American mtDNA sequences was obtained from Tom Parsons at the Armed Forces DNA Identification Laboratory. In this sample, mtDNAs with non-African haplotypes (five haplotype H, one haplotype J, and one haplotype U4) were excluded.

### Database assembly

A database of 3725 mtDNA HVS-I sequences from people living in sub-Saharan Africa was assembled from the published literature in October 2005 (Table 7) with the addition of 80 new mtDNA sequences from people belonging to the Malinke and Bambara ethnic groups in Mali (Table 8). DNA from these latter samples was isolated using a BuccalAmp DNA Extraction Kit (Epicentre, Madison, WI) from cheek swabs obtained from unrelated volunteers. MtDNA HVS-I sequences from two African-American population samples were then compared with these databases to determine how often individual HVS-I sequences are identical to African HVS-I sequences in the databases. For these comparisons, only sequences from 16030 to 16420 were considered, and both insertions and differences at positions 16182 and 16183 were ignored. In addition, a change to 16390A was inferred for all L2 haplogroup sequences that did not include this mutation. No attempt was made to correct any other errors that might be present among the published sequences. However, the presence of sequencing errors would have the effect of reducing the incidence of perfect matches so that the frequencies of perfect matches we observe should be considered minimum estimates. Matches to multiple individuals within an African ethnic group were considered a single match. Sequences included in the databases are available from Bert Ely.

## Authors' contributions

BE, JLW, BAJ participated in the assembly of the database. The database comparisons were performed by BE. All authors contributed to the interpretation of the data and the writing of the manuscript.

## Figures and Tables

**Table 1:** Definition of geographic regions.

Geographical regions	Historical areas	Major inclusive countries
West	Senegambia	Senegal, Gambia, northern Guinea
	Upper Guinea	Guinea, Sierra Leone, northwestern Liberia, parts of Mali
	Gold Coast	Ghana, Burkina Faso
	Bight of Benin	Western Nigeria, Benin
West Central	Bight of Bonny	Eastern Nigeria, western Cameroon
	Central Africa	Angola, the Congos, Equatorial Guinea, Gabon
South		Namibia, South Africa
Southeast	Mozambique	Mozambique, western Malagasy
East		Tanzania, Kenya, Uganda, Rwanda, Burundi, Ethiopia, Somalia, southern Sudan
North	Magrib	Morocco, Algeria, Spanish Sahara, Mauritania, Tunisia, Libya, Egypt, northern Sudan

**Table 2:** Characteristics of the sub-Saharan mtDNA HVS-I database.

Region	Region matched (%)							
	
	Total	Unique^a^	Multiple^b^	West	West Central	South	Southeast	East
West	1528	35	20	24	19	0	1	1
West Central	995	37	28	15	13	0	5	2
South	127	61	15	0	0	21	2	0
Southeast	416	25	51	1	15	1	7	0
East	659	59	12	1	2	0	0	27
Total	3725	40	24					

^a^Haplotypes found once or in a single ethnic group.

^b^Haplotypes found in ethnic groups from three or more regions.

**Table 3:** Number of perfect matches to African-American HVS-I sequences.

Number of matched ethnic groups	Sample	
	
	Gullah/Geechee	AFDIL
None	28	39
1	7	9
2–3	6	6
4–9	8	13
>9	25	30
Totals	74	97

**Table 4:** Imperfect matches to the Gullah/Geechee and AFDIL African-American HVS-I sequences.

	Number of sequences	Number of ethnic groups matched	Number of sequences
1 mismatch	42	1	12
		2–3	5
		4–9	15
		>9	10
>1 mismatch	25		ND

**Table 5:** Geographical source of mtDNA HVS-I matches.

Number of matches	Gullah/Geechee individuals			AFDIL African-American individuals		
	
	W. Africa	E. Africa	Both	W. Africa	E. Africa	Both
1–5	14	1	2	16	1	2
>5	5	0	24	5	0	33

**Table 6:** Distribution of single region matches.

Sample	West	W. Central	East
Gullah/Geechee	9	4	1
AFDL AA	7	3	1

**Table 7:** Mitochondrial DNA HVS-I sequences included in this study.

Ethnic group	Country	Sample size	Reference
**West Africa**			
Multiple	Senegal	50	Rando et al, 1998 [9]
Serer	Senegal	23	Rando et al, 1998 [9]
Wolof	Senegal	48	Rando et al, 1998 [9]
Mandenka	Senegal	110	Graven et al, 1995 [10]; Watson et al, 1997 [11]
Multiple groups	Guiné-Bissau	372	Rosa et al, 2004 [12]
Malinke	Mali	61	Ely et al, unpublished
Bambara	Mali	19	Ely et al, unpublished
Limba	Sierra Leone	67	Jackson et al, 2005 [3]
Loko	Sierra Leone	29	Jackson et al, 2005 [3]
Temne	Sierra Leone	121	Jackson et al, 2005 [3]
Mende	Sierra Leone	59	Jackson et al, 2005 [3]
Unknown group(s)	Sierra Leone	117	Monson et al, 2002 [13]
Fulbe	Nigeria, Niger	60	Watson et al, 1997 [11]
Hausa	Nigeria, Niger	20	Watson et al, 1997 [11]
Kanuri	Nigeria, Niger	14	Watson et al, 1997 [11]
Songhai	Nigeria, Niger	10	Watson et al, 1997 [11]
Tuareg	Nigeria, Niger	23	Watson et al, 1997 [11]
Yoruba	Nigeria	33	Vigilant et al, 1991 [14]; Watson et al, 1997 [11]
Unknown group(s)	Cabo Verde	292	Brehm et al, 2002 [15]
Total		1528	
**West Central Africa**			
Kotoko	Cameroon	18	Èerný et al, 2004 [16]
Hide	Cameroon	23	Èerný et al, 2004 [16]
Masa	Cameroon	31	Èerný et al, 2004 [16]
Mafa	Cameroon	32	Èerný et al, 2004 [16]
Bakaka	Cameroon	50	Coia et al, 2005 [17]
Bamileke	Cameroon	48	Coia et al, 2005 [17]
Bassa	Cameroon	46	Coia et al, 2005 [17]
Daba	Cameroon	20	Coia et al, 2005 [17]
Ewondo	Cameroon	53	Coia et al, 2005 [17]
Fali	Cameroon	41	Coia et al, 2005 [17]
Fulbe	Cameroon	34	Coia et al, 2005 [17]
Mandara	Cameroon	37	Coia et al, 2005 [17]
Podokwo	Cameroon	39	Coia et al, 2005 [17]
Tali	Cameroon	20	Coia et al, 2005 [17]
Tupuri	Cameroon	25	Coia et al, 2005 [17]
Uldeme	Cameroon	28	Coia et al, 2005 [17]
Biaka	Central African Republic	17	Vigilant et al, 1991 [14]; Watson et al, 1997 [11]
Mbenzele-Pygmy	Central African Republic	57	Destro-Bisol et al, 2004 [18]
Angolares	São Tomé and Príncipe	30	Trovoada et al, 2004 [19]
Forros	São Tomé and Príncipe	35	Trovoada et al, 2004 [19]
Tongas	São Tomé and Príncipe	38	Trovoada et al, 2004 [19]
Unknown group(s)	São Tomé and Príncipe	50	Mateu et al, 1997 [20]
Bubi	Equatorial Guinea	45	Mateu et al, 1997 [20]
Fang	Equatorial Guinea	11	Pinto et al, 1996 [21]
Mbuti	Democratic Republic of Congo	13	Vigilant et al, 1991 [14]; Watson et al, 1997 [11]
Bantu-speaking	Cabinda	110	Beleza et al, 2005 [4]
Mbundu	Angola	44	Plaza et al, 2004 [22]
Total		995	
**East Africa**			
Nuer	South Sudan	11	Krings et al, 1999 [23]
Dinka	South Sudan	47	Krings et al, 1999 [23]
Shilluk	South Sudan	7	Krings et al, 1999 [23]
Multiple groups	Ethiopia	21	Kivisild et al, 2004 [24]
Tigrais	Ethiopia, Eritrea	53	Kivisild et al, 2004 [24]
Gurage	Ethiopia	21	Kivisild et al, 2004 [24]
Afar	Ethiopia	16	Kivisild et al, 2004 [24]
Amhara	Ethiopia	120	Kivisild et al, 2004 [24]
Amhara	Ethiopia	7	Quintana-Murci et al, 1999 [25]
Oromo	Ethiopia	33	Kivisild et al, 2004 [24]
Oromo	Kenya, Ethiopia	18	Quintana-Murci et al, 1999 [25]
Unknown group(s)	Kenya	100	Brandstätter et al, 2004 [26]
Kikuyu	Kenya	24	Watson et al, 1997 [11]
Turkana	Kenya	37	Watson et al, 1997 [11]
Somali	Kenya, Somalia, Ethiopia	27	Watson et al, 1997 [11]
Hadza	Tanzania	17	Vigilant et al, 1991 [14]
Hadza	Tanzania	49	Knight et al, 2003 [27]
Dakota	Tanzania	18	Knight et al, 2003 [27]
Iraqw	Tanzania	12	Knight et al, 2003 [27]
Sukuma	Tanzania	21	Knight et al, 2003 [27]
Total		659	
**Southeast Africa**			
Multiple groups	Mozambique	109	Pereira et al, 2001 [6]
Multiple groups	Mozambique	307	Salas et al, 2002 [5]
Total		416	
**South Africa**			
!Kung	Botswana	34	Vigilant et al, 1991 [14]
!Kung	South Africa	43	Chen et al, 2000 [28]
Khwe	South Africa	31	Chen et al, 2000 [28]
Herero	Bostwana, Namibia	19	Vigilant et al, 1991 [14]
Total		127	

**Table 8:** Malinke and Bambara mitochondrial DNA HVS-I sequences included in this study.

ID	Ethnicity	Haplogroup	Hvs-I polymorphisms^a^
BAM676	Bambara	L1b	126 187 189 223 264 270 278 311
BAM612	Bambara	L1b1	126 187 189 223 256 264 270 278 293 311
BAM595	Bambara	L1b1	126 187 189 223 264 266 270 278 293 311
BAM599	Bambara	L1b1	126 187 189 223 264 266 270 278 293 311
BAM600-2	Bambara	L1b1	126 187 189 223 264 270 278 293 311
BAM060	Bambara	L2a	223 278 294 368 390
BAM598	Bambara	L2a1	189 192 209 223 278 294 309 390
BAM604	Bambara	L2a1a	223 278 286 294 309 390
BAM627	Bambara	L2b	114A 213 223 278 290 355 390
BAM659	Bambara	L2b1	114A 129 213 223 278 362 390
BAM037	Bambara	L2c	129 223 261 278 390
BAM685	Bambara	L2c2	183 223 264 278 320 390
BAM679-1	Bambara	L2c2	223 264 278 390
BAM629	Bambara	L2d2	111A 145 184 223 239 278 292 355 390 399 400
BAM068	Bambara	L3b	124 223 278 362
BAM072	Bambara	L3e2	223 284 320
BAM605	Bambara	L3e3	093 148 223 265 311
BAM027	Bambara	L3f1	049 129 209 223 292 295 311
BAM614	Bambara	L3f1	223 272 292 311
BAM 552	Malinke	L1b	111 126 187 189 223 239 270 278 311
BAM 237	Malinke	L1b	126 187 189 223 239 264 270 278 311
BAM 357	Malinke	L1b	126 187 189 223 239 264 270 278 311
BAM 040	Malinke	L1b	126 187 189 223 264 270 278 311
BAM 385	Malinke	L1b1	093 126 145 187 189 223 264 270 278 293 311
BAM 555	Malinke	L1b1	126 187 189 213 223 260 264 270 278 293 311
BAM 225	Malinke	L1b1	126 187 189 223 264 270 278 293 311 362 400
BAM 407	Malinke	L1c	129 189 215 223 278 294 311 360
BAM 013	Malinke	L1c2	015 15 bp ins 129 187 189 223 265 286 294 311 360
BAM 397	Malinke	L2a	189 192 223 278 294 390
BAM 221	Malinke	L2a	189 223 278 294 390
BAM 426	Malinke	L2a	223 278 286 294 390
BAM 083	Malinke	L2a	223 278 294 390
BAM 414	Malinke	L2a1	093 189 192 223 265 278 294 309 390
BAM 143	Malinke	L2a1	086 223 230 278 294 309 390
BAM 117	Malinke	L2a1	092 223 278 294 309 390
BAM 341	Malinke	L2a1	093 223 278 294 309 390
BAM 534	Malinke	L2a1	140 189 192 223 278 294 309 390
BAM 665	Malinke	L2a1	189 192 223 266 278 294 309 390
BAM 082	Malinke	L2a1	189 192 223 278 294 309
BAM 174	Malinke	L2a1	192 223 278 294 309 390
BAM 195	Malinke	L2a1	192 223 278 294 309 390
BAM 395	Malinke	L2a1	223 278 294 309 368 390
BAM 406	Malinke	L2a1	223 278 294 309 390
BAM 204	Malinke	L2a1	223 278 309 390
BAM 296	Malinke	L2b1	056 114A 129 213 223 278 362 390
BAM 085	Malinke	L2b1	093 114A 129 213 223 278 355 362 390
BAM 577	Malinke	L2b1	114A 129 213 223 278 311 355 362 390
BAM 290	Malinke	L2b1	114A 129 213 223 278 362 390
BAM 319	Malinke	L2b1	114A 129 213 223 278 362 390
BAM 401	Malinke	L2c	129 223 261 278 362 390
BAM 631	Malinke	L2c	162 223 261 278 390
BAM 427	Malinke	L2c	223 278 362 390
BAM 652	Malinke	L2c	223 278 390
BAM 269	Malinke	L2c1	223 256 261 278 318 390
BAM 432	Malinke	L2c2	093 223 264 278 362 390
BAM 151	Malinke	L2c2	223 264 278 390
BAM 680	Malinke	L2c2	223 264 278 390
BAM 681	Malinke	L2c2	223 264 278 390
BAM 187	Malinke	L2d1	014 129 278 300 354 390 399
BAM 110	Malinke	L2d2	111A 145 184 223 239 278 292 355 360 390 399 400
BAM 463	Malinke	L3b	124 223 278
BAM 185	Malinke	L3b	124 223 278 362
BAM 420	Malinke	L3b	124 223 278 362
BAM 430	Malinke	L3b	124 223 278 362
BAM 384	Malinke	L3b1	223 278 362
BAM 461	Malinke	L3d	111 124 223
BAM 521	Malinke	L3d	111 124 223
BAM 160	Malinke	L3d	124 223
BAM 375	Malinke	L3e2	172 223 239 320
BAM 402	Malinke	L3e2	172 223 320 353
BAM 467	Malinke	L3e2	188 223
BAM 525	Malinke	L3e2	188 223 320
BAM 041	Malinke	L3e2	223 257 290A 320
BAM 464	Malinke	L3e2	223 320
BAM 260	Malinke	L3e2	223 320 362
BAM 070	Malinke	L3f1	157 209 223 274 292 304 311
BAM 398	Malinke	L3f1	188 209 223 292 311
BAM 116	Malinke	L3f1	209 223 274 292 311
BAM 061	Malinke	U5	189 192 270 320
BAM 047	Malinke	U5	189 192 270 320

^a^Numbers indicate the position of differences from the Cambridge Reference Sequence minus 16,000. All mutations are transitions unless a letter designation is present.

**Table 9:** Gullah/Geechee mitochondrial DNA HVS-I sequences included in this study.

ID	Number	Hg	HvI polymorphisms
G299	1	A2	111 154 223 290 319 362
G211	2	B	93 182 183 189 217
RP22	1	K or H	189 265 311
G110	1	L0a1	129 148 168 172 187 188G 189 223 230 311 320
G207	1	L0a1	129 148 168 172 187 188G 189 223 230 293 320
G252	1	L1b1	114A 126 187 189 223 234 239 264 270 278 293 311
RP74	2	L1b1	126 187 189 223 264 270 278 293 311
RP287	1	L1b1	093 111 126 187 189 223 239 270 278 293 311 360
RP290	1	L1b1	111 126 187 189 223 239 270 278 293 311
RP93	1	L1b	126 189 223 264 270 278 311
RP291	1	L1c1a	129 187 189 223 274 278 293 294 311 360
RP25	3	L1c2	078 129 187 189 223 265C 286A 294T 311 320 360
G114	1	L1c	086 129 172 184 187 189 223 261 278 290 311 360
G124	1	L2a	183C 185 189 192 223 278 292 293 294 390
RP293	1	L2a	189 192 223 265 270 278 294 390
G260	1	L2a	189 192 223 278 294 390
RP313	1	L2a1	172 223 278 286 294 309 390
G158	1	L2a1	189 192 223 278 294 309 390
RP53	1	L2a1a	223 278 286 294 309 390
G326	1	L2a1a/b	092 223 278 286 290 294 309 327 390
G323	2	L2b	114A 129 212 213 223 278 390
G233	1	L2b	114A 129 213 223 274 278 390
G126	1	L2b	114A 129 213 223 278 390
G146	1	L2c	(A ins at 149) 207 223 242 278 390
RP94	1	L2c	051 223 278 390
G334	1	L2c	214 223 278 390
G349	1	L2c	214 223 278 390
RP298	1	L2c	223 278 311 390
G174	1	L2c	223 278 390
RP24	1	L2c	223 278 390
RP286	1	L2c2	223 264 278 390
G277	1	L2c2	148 264 278 311 390
G280	1	L2d1	093 129 172 189 207 278 300 354 390
RP59	1	L2d2	111A 145 184 223 239 278 292 311 355 390 399 400
RP26	1	L3b	124 182 183 189 223 278 362
G178	2	L3b	124 223 278 355 362
G173	1	L3b	124 223 278 362
G244	1	L3b2	124 223 278 311 362
G269	1	L3d	124 223
RP292	2	L3d	124 223 362
RP306	1	L3d3	051 124 223 278 304 311
RP295	2	L3e1	179 223 327
RP308	1	L3e1	207 223 327
G313	1	L3e1	223 327
RP302	1	L3e1	223 327 360
G337	1	L3e2	223 258 320
G172	2	L3e2	223 294 320
RP14	1	L3e2	223 320
G339	1	L3e2	223 320 399
G266	1	L3e2b	172 183C 189 223 278 320
G122	5	L3e2b	172 183C 189 223 320
RP28	2	L3e2b	172 189 223 320
RP45	1	L3e2b	189 223 320
G222	1	L3e3	093 223 265T
RP35	1	L3e3	189 223 265T 311
G199	2	L3e3	223 265T
G223	1	L3e4	051 093 209 223 264 320
RP294	1	L3f	209 223 311
G206	1	L3f1	129 209 223 292 295 311
G195	1	L3f1	129 209 223 292 295 311 368
G164	1	L3f1	129 209 223 292 311
G108	2	L3f1	209 223 292 311
Total	78		

## References

[B1] Salas A, Carracedo A, Richards M, Macaulay V (2005). Charting the ancestry of African Americans. Am J Hum Genet.

[B2] Jackson FL (2004). Human genetic variation and health: new assessment approaches based on ethnogenetic layering. Br Med Bull.

[B3] Jackson BA, Wilson J, Kirbah S, Sidney S, Rosenberger J, Bassie L, Alie JAD, McLean D, Garvey WT, Ely B (2005). Mitochondrial DNA genetic diversity among four ethnic groups in Sierra Leone. American Journal of Physical Anthropology.

[B4] Beleza S, Gusmao L, Amorim A, Carracedo A, Salas A (2005). The genetic legacy of western Bantu migrations. Hum Genet.

[B5] Salas A, Richards M, De la Fe T, Lareu MV, Sobrino B, Sanchez-Diz P, Macaulay V, Carracedo A (2002). The making of the African mtDNA landscape. Am J Hum Genet.

[B6] Pereira L, Macaulay V, Torroni A, Scozzari R, Prata MJ, Amorim A (2001). Prehistoric and historic traces in the mtDNA of Mozambique: insights into the Bantu expansions and the slave trade. Ann Hum Genet.

[B7] Phillipson D (1993). African Archaeology.

[B8] Sengova J, Yelvington KA (2006). "My mother dem nyum to plan' reis": Reflections on Gullah/Geechee Creole communication, connections, and the construction of cultural identity. Afro-Atlantic Dialogues: Anthropology in the Diaspora.

[B9] Rando JC, Pinto F, Gonzalez AM, Hernandez M, Larruga JM, Cabrera VM, Bandelt HJ (1998). Mitochondrial DNA analysis of northwest African populations reveals genetic exchanges with European, near-eastern, and sub-Saharan populations. Ann Hum Genet.

[B10] Graven L, Passarion G, Ornella S, Boursot P, Santachiara-Benerecetti S, Langaney A, Excoffier L (1995). Evolutionary correlation between control region sequence and restriction polymorphisms in the mitochondrial genome of a large Senegalese Mandenka sample. Mol Biol Evol.

[B11] Watson E, Forster P, Richards M, Bandelt HJ (1997). Mitochondrial footprints of human expansions in Africa. Am J Hum Genet.

[B12] Rosa A, Brehm A, Kivisild T, Metspalu E, Villems R (2004). MtDNA profile of West Africa Guineans: towards a better understanding of the Senegambia region. Ann Hum Genet.

[B13] Monson KL, Miller KWP, Wilson MR, Dizinno JA, Budowle B (2002). The mtDNA population database: an intergrated software and database resource for forensic comparison. Forensic Science Coomunications.

[B14] Vigilant L, Stoneking M, Harpending H, Hawkes K, Wilson AC (1991). African populations and the evolution of human mitochondrial DNA. Science.

[B15] Brehm A, Pereira L, Bandelt HJ, Prata MJ, Amorim A (2002). Mitochondrial portrait of the Cabo Verde archipelago: the Senegambian outpost of Atlantic slave trade. Ann Hum Genet.

[B16] Cerny V, Hajek M, Cmejla R, Bruzek J, Brdicka R (2004). mtDNA sequences of Chadic-speaking populations from northern Cameroon suggest their affinities with eastern Africa. Ann Hum Biol.

[B17] Coia V, Destro-Bisol G, Verginelli F, Battaggia C, Boschi I, Cruciani F, Spedini G, Comas D, Calafell F (2005). Brief communication: mtDNA variation in North Cameroon: Lack of Asian lineages and implications for back migration from Asia to sub-Saharan Africa. Am J Phys Anthropol.

[B18] Destro-Bisol G, Coia V, Boschi I, Verginelli F, Caglia A, Pascali V, Spedini G, Calafell F (2004). The analysis of variation of mtDNA hypervariable region 1 suggests that Eastern and Western Pygmies diverged before the Bantu expansion. Am Nat.

[B19] Trovoada MJ, Pereira L, Gusmao L, Abade A, Amorim A, Prata MJ (2004). Pattern of mtDNA variation in three populations from Sao Tome e Principe. Ann Hum Genet.

[B20] Mateu E, Comas D, Calafell F, Perez-Lezaun A, Abade A, Bertranpetit J (1997). A tale of two islands: population history and mitochondrial DNA sequence variation of Bioko and Sao Tome, Gulf of Guinea. Ann Hum Genet.

[B21] Pinto F, Gonzalez AM, Hernandez M, Larruga JM, Cabrera VM (1995). Genetic relationship between the Canary Islanders and their African and Spanish ancestors inferred from mitochondrial DNA sequences. Ann Hum Genet.

[B22] Plaza S, Salas A, Calafell F, Corte-Real F, Bertranpetit J, Carracedo A, Comas D (2004). Insights into the western Bantu dispersal: mtDNA lineage analysis in Angola. Human Genetics.

[B23] Krings M, Salem AE, Bauer K, Geisert H, Malek AK, Chaix L, Simon C, Welsby D, Di Rienzo A, Utermann G (1999). mtDNA analysis of Nile River Valley populations: A genetic corridor or a barrier to migration?. Am J Hum Genet.

[B24] Kivisild T, Reidla M, Metspalu E, Rosa A, Brehm A, Pennarun E, Parik J, Geberhiwot T, Usanga E, Villems R (2004). Ethiopian mitochondrial DNA heritage: tracking gene flow across and around the gate of tears. Am J Hum Genet.

[B25] Quintana-Murci L, Semino O, Bandelt HJ, Passarino G, McElreavey K, Santachiara-Benerecetti AS (1999). Genetic evidence of an early exit of Homo sapiens sapiens from Africa through eastern Africa. Nat Genet.

[B26] Brandstatter A, Peterson CT, Irwin JA, Mpoke S, Koech DK, Parson W, Parsons TJ (2004). Mitochondrial DNA control region sequences from Nairobi (Kenya): inferring phylogenetic parameters for the establishment of a forensic database. Int J Legal Med.

[B27] Knight A, Underhill PA, Mortensen HM, Zhivotovsky LA, Lin AA, Henn BM, Louis D, Ruhlen M, Mountain JL (2003). African Y chromosome and mtDNA divergence provides insight into the history of click languages. Curr Biol.

[B28] Chen Y, Olckers A, Schurr T, Kogelnik A, Huoponen K, Wallace D (2000). mtDNA Variation in the South African Kung and Khwe – and their genetic relationships to other African populations. Am J Hum Genet.

